# Subunit Mobility and the Chaperone Activity of Recombinant αB-Crystallin

**DOI:** 10.2174/1874091X00802010116

**Published:** 2008-09-02

**Authors:** A Krushelnitsky, N Mukhametshina, Y Gogolev, N Tarasova, D Faizullin, T Zinkevich, O Gnezdilov, V Fedotov

**Affiliations:** 1 Kazan Institute of Biochemistry and Biophysics, Russian Academy of Sciences, Kazan, Russia; 2 Kazan Physical-Technical Institute, Russian Academy of Sciences, Kazan, Russia

## Abstract

The comparison of the chaperone-like activity of native and covalently cross-linked human αB-crystallins has confirmed the important role of the subunit mobility in the chaperoning mechanism. Our data clearly demonstrate that the chaperone-like activity of α-crystallin is not only a surface phenomenon as was suggested by some researchers.

## INTRODUCTION

α-Crystallin is a major protein constituent of the vertebrate eye lens. It is responsible for maintaining lens transparency by preventing the uncontrolled aggregation of other proteins, mainly β- and γ-crystallins. This protein was found in other tissues as well, and now there is a little doubt that age-related post-translational modifications of α-crystallin decreasing its chaperone activity may cause not only cataract but also some other serious diseases [1-4]. Native α-crystallin consists of two types of homologous subunits, αA- and αB-crystallins, with molecular mass ~20 kDa each. They form oligomeric aggregates with a wide distribution of molecular mass from 300 kDa to 1.2 MDa and variable quaternary structure [5]. It is known that homooligomers consisting of only αA or αB crystalline subunits have similar properties to the native heterooligomers and also reveal chaperone-like activity [6]. Being studied quite intensively over the last few decades, α-crystallin still remains one of the most interesting and unknown proteins for molecular biophysicists and biochemists, see reviews [1,3,7,8]. The main reason for that is the unknown spatial structure of α-crystallin: no one could crystallize this protein so far, and thus the X-ray analysis could not be applied. Furthermore, the molecular mass of α-crystallin is too high for the structure determination by liquid-state NMR methods. The structures of α-crystallin - target protein complexes are not known either. Without knowledge of the structure, the detailed molecular mechanisms of the chaperone-like activity of α-crystallin are still to a great extent a subject of hypotheses and speculations.

One of the issues being intensively discussed is the mechanism of binding of α-crystallin to target proteins. By now it is known quite well that the quaternary structure of α-crystallin is rather mobile, and subunits exchange between α-crystallin aggregates [9-1]. Many researchers believe that the subunit mobility is directly involved in the binding process of α-crystallin to target proteins since there is a correlation between subunits exchange and the chaperone-like activity [12-15]. It is supposed that target proteins get inside the α-crystallin aggregate, and this obviously would be impossible were the α-crystallin subunits immovable with respect to each other [10]. There are indications that binding target protein to α-crystallin is accompanied by structural rearrangements of α-crystallin subunits, which also would not be possible without subunit mobility [16]. On the other hand, few years ago Augusteyn [17] presented a direct experimental indication that the subunit mobility is not important for the chaperone-like activity and that binding target proteins to α-crystallin is only a surface phenomenon: being cross-linked by glutaraldehyde, α-crystallin reveals even somewhat increased chaperone-like activity preventing thermally induced aggregation of β-crystallin. Similar results were obtained for the Hsp26 [18]. Thus, there is a direct contradiction between various types of data, which is not resolved yet. The aim of the present work is to find out whether the results of the works [17] and [18] have a general nature, or these are only specific cases. It is very well known that depending on the source of α-crystallin (extracted from eyes or recombinant), the subunit composition (αA and αB), type of the target protein, posttranslational modifications (phosphorylation, chain truncation, etc.), the chaperone-like activity of α-crystallin may differ significantly. The above may also affect the role of the subunit dynamics in the chaperoning mechanism. To check this, we have compared the chaperone-like activity of the intact and cross-linked recombinant αB-crystallin. In contract to [17,18], our data clearly demonstrate that the subunit dynamics is an essential factor of the chaperone activity mechanism.

## MATERIALS AND METHODS 

### Expression and Purification of Human αB-Crystallin

*E. coli* strain BL21(DE3) was transformed with expression constructs peT16b-αB according to the protocol described in [19]. A colony was picked out from the freshly streaked plate and inoculated with 50 ml of LB medium containing ampicillin (50 mg/ml). The culture was grown to log phase (A_590_, 1.5–1.7). A 25-ml volume of the log phase culture was inoculated into 1 liter of LB containing ampicillin (50 mg/ml). The culture was incubated at 37 ^°^C with vigorous shaking until the optical density reached 0.4 – 0.6 at 590 nm, then the expression was induced by the addition of 1 ml of 1 M isopropyl β-D-1-thiogalactopyranoside. Incubation continued for another 6 h. Cells were then harvested by centrifugation at 8,000 g for 10 min and kept at – 80 °C

The recombinant protein was purified by the combination of ammonium sulfate fractionation and Q-Sepharose anion-exchange FPLC (Pharmacia, Sweeden). The collected cells were suspended in TE buffer (20 mM Tris-HCl, 0.5 mM EDTA, pH 8.5) at a ratio of 3 ml of buffer to 1 g (wet weight) of cells; 100mM phenylmethylsulfonyl fluoride (4μl per g of cells) and lysozyme (0.1 mg per g of cells) were added. The mixtures were kept at 4 ^º^C for 20 min, after which deoxycholic acid (4 mg/g of cells) was added. After thorough mixing, DNase (2μl, 10 mg/ml) was added, and incubation at 37 °C was continued until the mixture was no longer viscous. The clear cell lysate was obtained after centrifugation at 12,500 g for 30 min and then was fractionated by the addition of solid ammonium sulfate up to 30% final concentration (w/v). After centrifugation at 17,000 g for 20 min, the supernatant was saturated up to 60% of ammonium sulfate. The resulting precipitate was resuspended in phosphate buffer (pH 6.9) and dialyzed against the same buffer with two buffer changes. The dialyzed sample was loaded onto Q-Sepharose 10/15 column equilibrated in TE buffer (pH 6.9). The unbound proteins containing αB-crystallin were collected and dialyzed overnight against TE buffer (pH 8.5). The dialyzed sample was loaded onto Q-Sepharose 10/15 column equilibrated in the same buffer. The column was washed until unbound proteins were not detected. The elution was achieved using a linear gradient of 0 – 1 M NaCl at a flow rate of 1 ml/min. The elution of proteins was monitored by the optical absorbance at wavelength 280 nm.

**Fig. (1):**
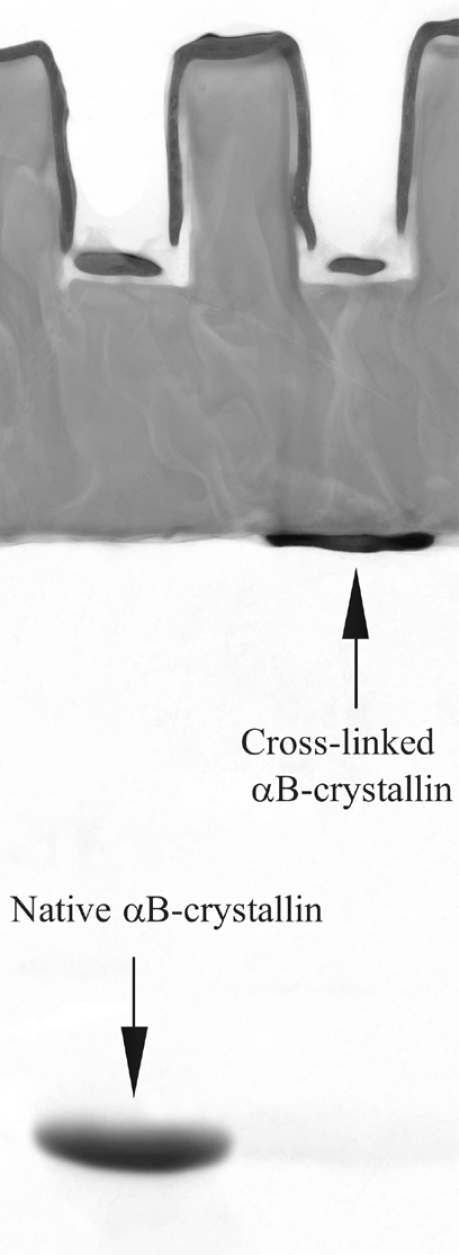
Electrophoresis in polyacrylamide gel with sodium dodecyl sulfate (SDS-PAGE) of the native and cross-linked αB-crystallins.

Cross-linking was performed according to the protocol used in [17]. αB-crystallin (0.05 mM) was incubated in phosphate buffered saline (PBS, 20 mM phosphate buffer, 150 mM NaCl, pH 7.2) with 0.3 mM glutaraldehyde at 20 ºC for 30 h. Then, the protein was dialyzed against PBS. The native and cross-linked samples were examined by electrophoresis. Only residual traces of the monomeric subunits could be observed in the cross-linked sample, see Fig. (1). This means that practically all subunits in the cross-linked sample are covalently linked within aggregates. Thus, the subunit mobility in this sample is suppressed. Target proteins (hen egg white lysozyme and alcohol dehydrogenase) were purchased from Sigma.

### Determination of the Chaperone-Like Activity of αB-Crystallin

The activity was examined by the ability of αB-crystallin to inhibit the aggregation of two target proteins: hen egg white lysozyme and alcohol dehydrogenase. The lysozyme aggregation was induced by addition of 20 mM dithiothreitol (DTT) to the protein solution [16]. The aggregation of alcohol dehydrogenase was achieved by rising the temperature of the solution up to 48 ºC [20]. The protein aggregation process was monitored by the optical absorbance at wavelength 360 nm on Perkin-Elmer Lambda-25 spectrophotometer.

### Fourier-Transformed Infra-Red (FTIR) Spectroscopy Measurements

Spectra were recorded on Vector 22 spectrophotometer (Bruker) in the range of 4000 - 1000 cm^-1^ with resolution of 4 cm^-1^. αB-crystallin was measured in D_2_O solutions to remove the H_2_O absorbance overlapping the Amide I peak. Protein concentration was 1.5-2% by weight. The solutions were placed in CaF_2_ cell with 100 micron path length. Spectra were recorded at 25^o^C. Spectra of pure D_2_O were subtracted from the corresponding original spectra.

### Translational Self-Diffusion Coefficient (SDC) Measurements

SDC’s were measured using the standard NMR stimulating echo pulse sequence with pulsed field gradient [21]. Experiments were performed on the Bruker Avance NMR spectrometer operating at the resonance frequency 400 MHz for protons. Protein concentration in solution was 3% by weight for both native and cross-linked αB-crystallins. The SDC was defined from the fitting the diffusion decays (the dependence of the signal intensity on the magnetic field gradient) according to the standard formula:

$I(g)=A\cdot \exp\left(-\gamma^{2}g^{2}\delta^{2}{\mathrm{DT}}_{d}\right)$

where *g* is the magnetic field gradient, *A* is the normalization coefficient, *γ* is the gyromagnetic ratio for protons, *δ* is the duration of the field gradient pulses, *T_d_* is the diffusion time, *D* is the SDC. The integral intensity over the whole proton spectrum (except the peak corresponding to water protons at 4.7 ppm) was analyzed in the diffusion experiments. Diffusion time (*T_d_*) was 70 ms, duration of the gradient pulses (*δ*) was 5 ms. The experiments were carried out at ambient temperature (23-25 ºC) without temperature stabilization. The experimental error of the SDC estimation was 5-7 %.

## RESULTS AND DISCUSSION

To relate the difference in the chaperone-like activity between native and cross-linked αB-crystallins solely to the subunit mobility effect, one has to exclude other possible reasons of such a difference. Namely, cross-linking should not lead to the change of the protein structure and appearance of inter-molecular (i.e. inter-aggregate) cross-links. The size of the cross-linked aggregates was checked by Augusteyn by means of the gel permeation chromatography [17]. In our work, we used other experimental techniques to present an independent proof that cross-linking does not change α-crystallin properties, except subunit mobility. We have recorded IR spectra and measured translational SDC of the native and cross-linked αB-crystallins. Fig. (**2**) presents IR spectra of the Amide I peak that is very sensitive to the protein secondary structure composition. It is seen that native and cross-linked proteins reveal no secondary structure differences. Hence, the structural effect on the chaperone-like activity can be ruled out. 

Fig. (**3**) presents the diffusion decays for the native and cross-linked αB-crystallins. The SDC values correspond quite well to the previous light scattering self-diffusion measurements of α-crystallin [22]. Note that the diffusion decays could be well approximated by a single SDC. It is known that SDC is proportional to the linear size of a Brownian particle, hence, SDC~M^0.33^, where M is a molecular weight. Thus, the shape of the diffusion decays is not very sensitive to molecular mass distributions. However, our data indicate that the molecular mass distribution of the αB-crystallin molecules is at least not very wide. So, two independent checks performed by Augusteyn [17] and by us confirm that glutaraldehyde cross-linking does not change the size of α-crystallin oligomers.

Figs. (**4** and **5**) present the measurements of the chaperone-like activity of αB-crystallin with hen egg white lysozyme and alcohol dehydrogenase as target proteins, respectively. It is clearly seen that the cross-linking essentially suppresses the αB-crystallin activity in comparison with the native αB-crystallin. These data are an indication of the essential role of the subunit mobility in the chaperoning mechanism of recombinant αB-crystallin and support many previous works on this topic cited in the Introduction. Thus, the chaperone activity is not always only a surface phenomenon as it is supposed in [17]. Our data nevertheless demonstrate that some surface effect does also exist since the cross-linked αB-crystallin sample reveals some activity as well. 

Few possible reasons can be responsible for the discrepancy between our and Augusteyn’s results. These are: 1) using recombinant and extracted from lens proteins (in the latter case various post-translational modifications are possible); 2) different subunit composition; 3) different target proteins used in these studies. It is likely that depending on the preparations and conditions of the experiment, surface binding of target proteins and their binding inside α-crystallin oligomers may have different prevalence. To find out, how all these factors may affect the importance of the subunit dynamics in the chaperoning mechanism, much more extensive set of the experiments is needed which is outside the scope of this report. However, we think that the difference between the extracted from lenses and recombinant α-crystallins is the most probable reason explaining the discrepancy between our results and those presented in [17]. Note that Liu and co-workers [10] also supposed that this difference is a likely explanation of the contradiction between their and Augusteyn’s results. The difference between target proteins and subunit composition seems to us less important since 1) we used in our study two different target proteins which demonstrated similar results and 2) the chaperoning mechanisms of αA/αB and αB-crystallins are hardly very different. However, these are mere suppositions, while the available data do not allow making definite conclusions. Nevertheless, our data clearly demonstrate that the conclusion on the surface nature of the chaperoning mechanism of α-crystallin is, at least, generally incomplete.

## Figures and Tables

**Fig. (2):**
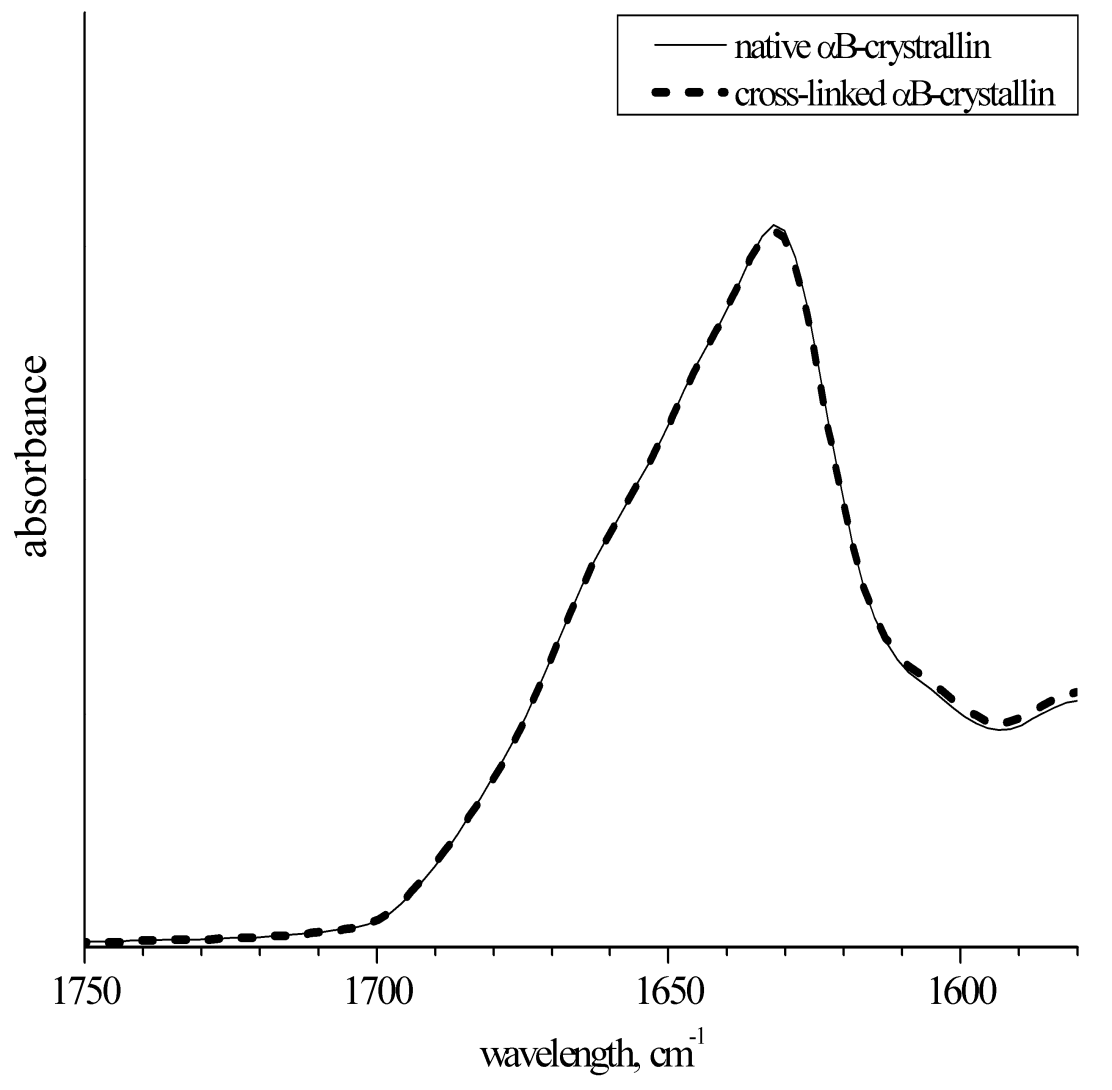
The Amide I band of the infra-red spectra of the native and cross-linked αB-crystallin. The spectra were normalized to the integral intensity of the Amide I band

**Fig. (3):**
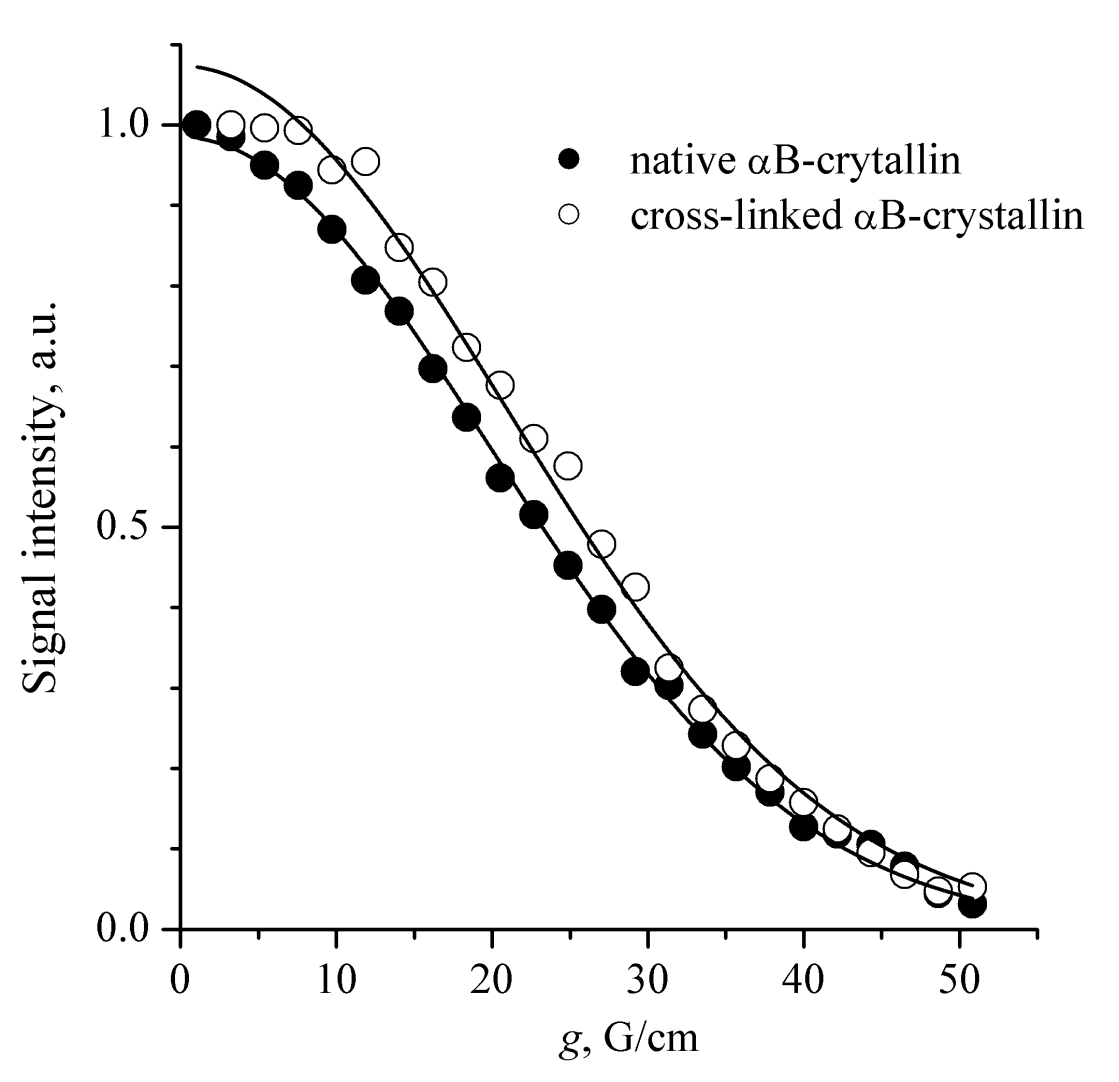
Diffusion decays for the native and cross-linked αB-crystallins. Solid lines are the fitting curves plotted according to Eq. (1). The SDC are 2.6×10^-11^ and 2.4×10^-11^ m^2^/s for the native and cross-linked αB-crystallins, respectively

**Fig. (4):**
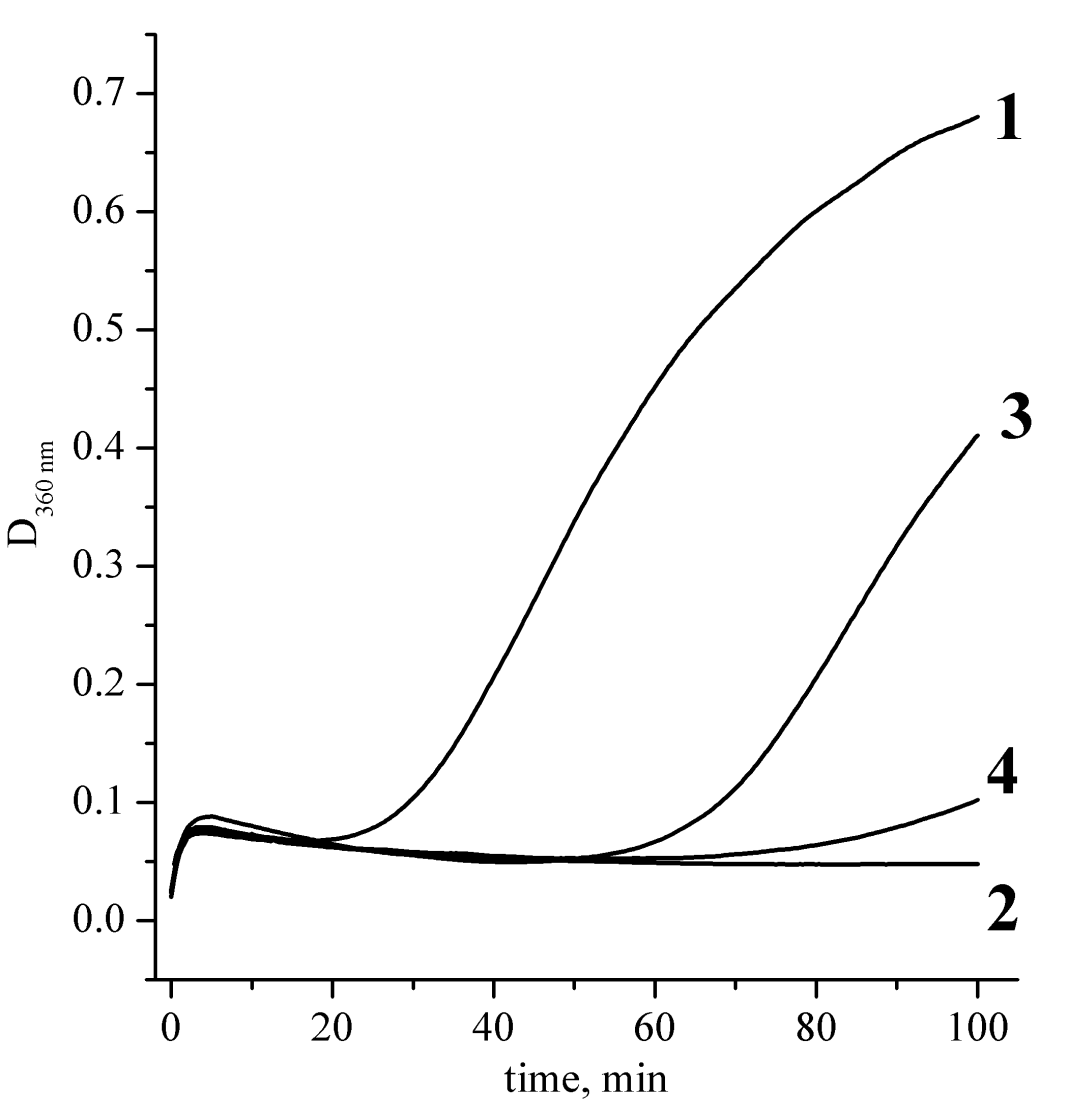
Aggregation kinetics of hen egg white lysozyme solutions after addition of DTT. Lysozyme concentration was always 0.1 mg/ml, experiments were performed at 37 ºC, phosphate buffer, pH 7.0. Curves definition: 1 – pure lysozyme solution; 2 – lysozyme and native αB-crystallin at concentration 0.1 mg/ml; 3 – lysozyme and cross-linked αB-crystallin at concentration 0.1 mg/ml; 4 – ly-sozyme and cross-linked αB-crystallin at concentration 0.2 mg/ml

**Fig. (5):**
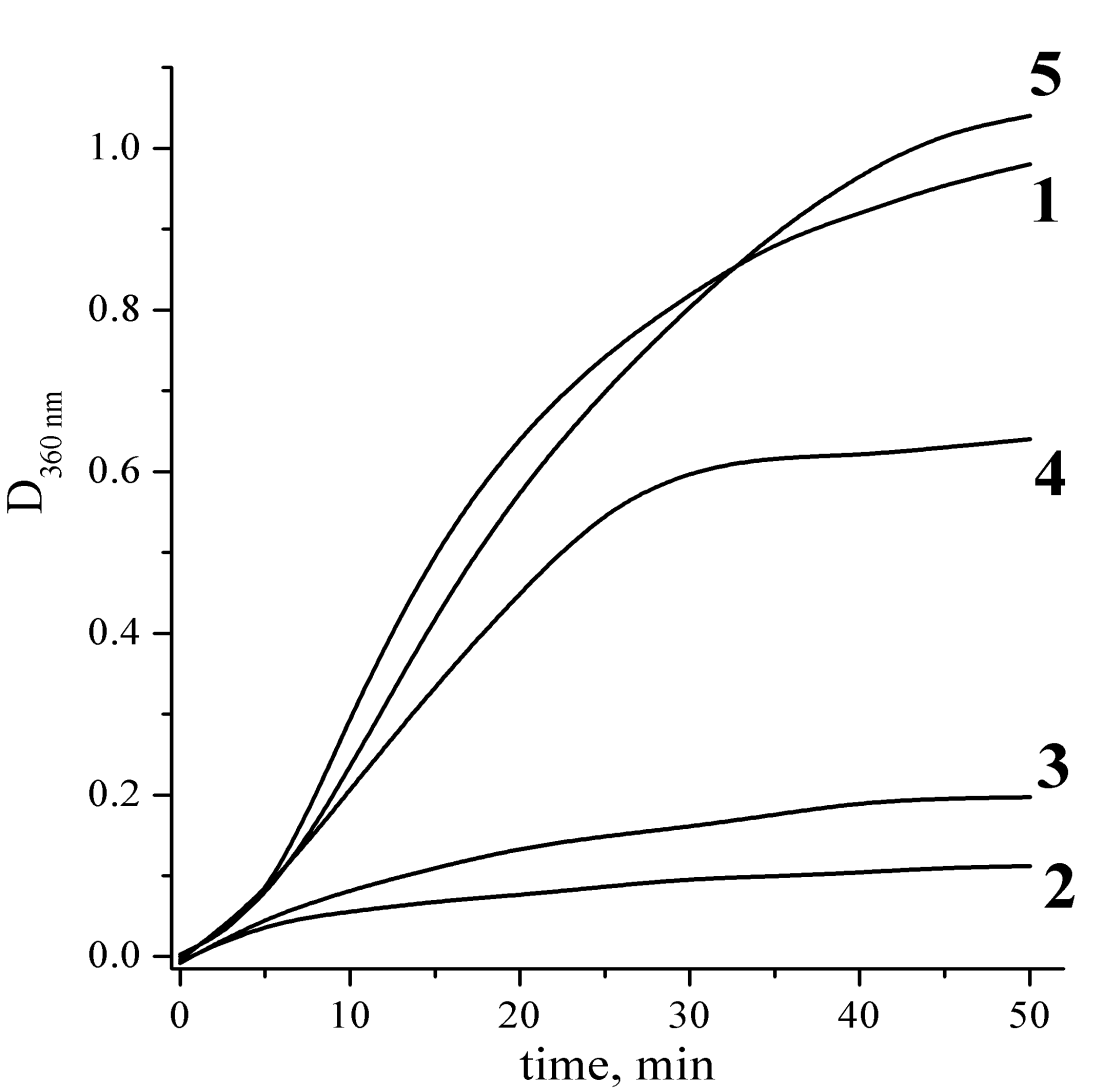
Aggregation kinetics of alcohol dehydrogenase (ADG) at 48 ^º^C. Alcohol dehydrogenase concentration was always 0.5 mg/ml, phosphate buffer, pH 7.0. Curves definition: 1 – pure ADG solution; 2 – ADG and native αB-crystallin at concentration 0.2 mg/ml; 3 – ADG and native αB-crystallin at concentration 0.05 mg/ml; 4 – ADG and cross-linked αB-crystallin at concentration 0.2 mg/ml; 5 – ADG and cross-linked αB-crystallin at concentration 0.05 mg/ml
